# “Entre Nosotras:*”* a qualitative study of a peer-led PrEP project for transgender latinas

**DOI:** 10.1186/s12913-023-09707-x

**Published:** 2023-09-20

**Authors:** Sophia Zamudio-Haas, Kim Koester, Luz Venegas, Ariana Salinas, Cinthya Herrera, Luis Gutierrez-Mock, Layla Welborn, Madeline B. Deutsch, Jae Sevelius

**Affiliations:** 1 Department of Medicine, University of California, 550 16th Street, 3rd Floor, CA 94158 San Francisco, USA; 2 La Clinica de La Raza, 3451 East 12th Street, CA 94601 Oakland, USA

**Keywords:** Transgender health, PrEP, HIV services, Peer navigation, Trans women, Peer support services, Healthcare equity, Health disparities

## Abstract

**Background:**

Uptake of HIV pre-exposure prophylaxis (PrEP) remains low among transgender people as compared to other subgroups, despite high rates of HIV acquisition. In California, Latinx people comprise 40% of the population and Latina transgender women experience some of the highest burden of HIV of any subgroup, indicating a critical need for appropriate services. With funding from the California HIV/AIDS Research Programs, this academic-community partnership developed, implemented, and evaluated a PrEP project that co-located HIV services with gender affirming care in a Federally Qualified Heath Center (FQHC). Trans and Latinx staff led intervention adaptation and activities.

**Methods:**

This paper engages qualitative methods to describe how a PrEP demonstration project- Triunfo- successfully engaged Spanish-speaking transgender Latinas in services. We conducted 13 in-depth interviews with project participants and five interviews with providers and clinic staff. Interviews were conducted in Spanish or English. We conducted six months of ethnographic observation of intervention activities and recorded field notes. We conducted thematic analysis.

**Results:**

Beneficial elements of the intervention centered around three intertwined themes: creating trusted space, providing comprehensive patient navigation, and offering social support “entre nosotras” (“between us women/girls”). The combination of these factors contributed to the intervention’s success supporting participants to initiate and persist on PrEP, many of whom had previously never received healthcare. Participants shared past experiences with transphobia and concerns around discrimination in a healthcare setting. Developing trust proved foundational to making participants feel welcome and “en casa/ at home” in the healthcare setting, which began from the moment participants entered the clinic and continued throughout their interactions with staff and providers. A gender affirming, bilingual clinician and peer health educators (PHE) played a critical part in intervention development, participant recruitment, and patient navigation.

**Conclusions:**

Our research adds nuance to the existing literature on peer support services and navigation by profiling the multifaced roles that PHE served for participants. PHE proved instrumental to empowering participants to overcome structural and other barriers to healthcare, successfully engaging a group who previously avoided healthcare in clinical settings.

## Background

Transgender women continue to have the highest levels of new HIV infections and HIV prevalence of any subgroup, both in the US and globally [1, 2]. While representing a small proportion of the overall population, they nonetheless shoulder a disproportionate burden of HIV [3–5]. Social and structural factors rooted in transphobia and discrimination against transgender (“trans”) people create the context for high HIV risk and reduce access to health care [6–9]. Structural factors - such as lack of access to gender affirming care [10, 11], high levels of unemployment [10], lack of stable housing [10], self-medication via non-prescription drug [12]- have all been well documented as contributors to elevated HIV prevalence for trans women.

Despite high rates of HIV acquisition, to date uptake of HIV pre-exposure prophylaxis (PrEP) has been low among trans populations as compared to other subgroups [13]. Among a national sample of trans people surveyed about PrEP, 70% met CDC criteria for PrEP eligibility, yet less than 10% of trans women respondents reported ever being prescribed PrEP [13]. Of Latinx^1^ - a gender inclusive term for people of Latin American or Caribbean origin living in the U.S [14]- people in the U.S. who could benefit from PrEP, only 14% were prescribed PrEP in 2019 [15]. This disparity takes on critical importance in California, where Latinx people comprise about 40% of California’s population [16], yet Latina trans women, along with African American trans women, experience some of the highest rates of HIV of any subgroup in the state, indicating a critical need for gender-affirming and population-specific prevention services [17, 18]. Research has shown that while many Latina trans women are willing to take PrEP, uptake among Latina trans women remains low [19]. Knowledge gaps and concerns about potential drug-drug interactions between PrEP and have been cited as contributors to low levels of uptake among trans women generally [20], and among Latina trans women specifically [20].

Implementation science literature has begun to unpack the multifaced determinants that contribute to low PrEP uptake and persistence among trans women [21]. Structural factors such as low access to health care can force choices to prioritize gender affirming care such as hormone therapy over preventative care like PrEP [11, 22]. Several studies have shown the ways that program level determinants, such as health communication and promotional strategies that group trans women erroneously with cisgender men who have sex with men can alienate trans women and create the perception that PrEP is not for them [21, 11]. There have been important steps forward, often led by trans people for trans people, to develop communication strategies and networks of trans specific programs and services [23, 24].

California offers a unique social and political environment that protects the rights of immigrants and trans people, including a robust social services network; [25–27]nonetheless, there is opportunity for more intersectional services for Latinx trans people. While services exist in California to meet health care needs of Spanish speaking immigrants and other Latinx people, few agencies provide trans specific services, such as gender affirming care [17, 28]. On the other hand, clinics and agencies that support trans health and offer trans specific programming might not have the expertise in immigration assistance or employ Spanish speaking staff [29]. California and other areas with substantial Spanish speaking trans populations need to bolster culturally appropriate, gender affirming services that address the unique considerations for this important group [17].

To address this programmatic gap, researchers at the University of California, San Francisco (UCSF) in collaboration with two community clinical partners, La Clínica de La Raza (“La Clinica”) in Oakland and Gender Health Center (GHC) in Sacramento, developed a trans-specific PrEP demonstration project called Trans Research-Informed Communities United in Mobilization for the Prevention of HIV (TRIUMPH), funded by the California HIV/AIDS Research Program (CHRP). TRIUMPH was a community-led, trans-specific PrEP demonstration project that documented high levels of PrEP initiation in a young transgender and gender diverse cohort at risk of HIV acquisition, with high levels of retention among Latinx participants [30]. Core components of the two-site demonstration project included employing peer health educators (PHE), engaging in community mobilization, and conducting monthly groups [30]. At each site, demonstration project teams adapted programming slightly to align with the demographics of trans communities in each service area.

Here we explore the mechanisms by which the demonstration project team at the La Clínica site implemented TRIUMPH, where the site-specific implementation was referred to as Triunfo (the Spanish translation for TRIUMPH). Drawing on in-depth interviews with staff and participants, as well as ethnographic observations conducted during intervention services, we present qualitative findings to describe how the La Clinica site succeeded in engaging a marginalized group and helped participants overcome formidable structural barriers to ongoing care [21]. Our findings provide guidance for other programs to successfully provide PrEP and other sexual health care, co-located with gender affirming care to Latina trans women.

## Methods

### Aim and study design

This qualitative inquiry aimed to understand the experiences of staff and participants in an innovative PrEP demonstration project for transgender women, that combined sexual health services including HIV prevention and PrEP with gender affirming care. The study relied on qualitative in-depth interviews with participants and staff as well as ethnographic observation.

### Study setting

La Clínica de la Raza (hereafter, “La Clínica”) is a Federally Qualified Health Center (FQHC), offering a range of services driven by a culturally informed commitment to community wellness. La Clinica has 30 service sites around the Bay Area’s Alameda, Contra Costa, and Solano counties. In 2019, La Clinica provided services to 91,000 patients, 64% of whom were Latinx [31]. The patient population is predominantly low income, with 91% living below 200% of the federal poverty level [31]. La Clincia service sites vary in scope of services offered and populations served; among these are primary care facilities, youth school-based sites, mobile clinics, optical, and dental centers. La Clinica Fruitvale Village, the Triunfo implementation site, is the largest of the service sites, providing comprehensive primary care, dental, preventative services, social services, pharmacy, and a full-service lab.

### Intervention description

TRIUMPH was a two-site demonstration project, implemented at La Clínica (‘Triunfo’) and GHC. Each site implemented similar key components: (1) Services from peer health educators (PHE) who led all project activities and provided PrEP education, navigation, and social support; (2) Drop-in clinics where patients could access hormone therapy and PrEP services from primary care providers (3) Monthly groups that offered a social space and facilitated discussions focused on a range of health related topics; and (4) Community mobilization and PrEP champions, including community events and an annual selected PrEP champion. These efforts focused on increasing community-level PrEP awareness, increasing empowerment around HIV prevention, and decreasing stigma around PrEP. From October 2017 to March 2020, TRIUMPH enrolled 185 transgender and gender diverse participants; 77 of these participants were enrolled at La Clinica [30].

This analysis focuses on the implementation of Triunfo at La Clínica. La Clinica staff (including the HIV and gender-affirming care clinician who is a family nurse practitioner), and the project director and PHEs (all three trans Latinx people), implemented cultural adaptations to tailor the intervention to the specific needs of the predominantly Spanish-speaking clients. These adaptations included: providing navigation to appointments related to immigration such as legal clinics or visits with immigration lawyers, linking monthly groups to culturally important celebrations, serving typical Latin American dishes such as tamales and pan dulce at the drop-in clinics, and linking Triunfo-sponsored community events to culturally important holidays such as the ‘PrEP Posada’ held before the December holiday. The intervention core components are described below in Table 1. With implementation of this unique programming, La Clínica sought to bring in trans women, a population that staff recognized as having unmet health needs and previously not a significant part of the range of Latinx patients served. While La Clínica previously provided services to a few trans patients, Triunfo was their first trans-specific programming.

**Table 1:** Triunfo Intervention Core Components

**General Clinic**	La Clinica de la Raza is a Federally Qualified Health center located in Oakland, California. It provides a wide range of medical, dental, and behavioral health services to diverse and underserved Latinx communities, including low-income families, immigrants, and people experiencing homelessness.
**Drop-in Clinic**	The drop-in clinic was located on the urgent care floor of the clinic, specifically designated for gender-affirming delivery of care days. The clinic provided a private room, for gender-diverse patients to wait (should they choose to), anchored around a table, with chairs, and a bowl of condoms and lubricant. The private room allowed patients to engage in open dialogue with one another as they waited to be seen by the clinician.
**Gender-affirming Clinician**	The clinician was a well-known bilingual gender-affirming Family Nurse Practitioner with extensive expertise in both trans-specific and HIV care and had an excellent reputation for providing supportive patient-centered care to trans communities.
**Patient Navigation**	Two peer health educators (PHEs), who were well-known transgender women within the community, followed up with both new and existing patients to schedule their initial clinical and follow-up visits. The PHEs physically accompanied participants between clinical departments- registration, laboratory, and pharmacy- introducing them to the clinical staff in each department along the way. PHEs additionally conducted outside of clinic navigation, as needed, brining participants to legal appointments, supporting name change applications at Department of Motor Vehicles, and giving rides to other gender affirming services not offered through La Clinica.
**Monthly Groups**	The PHEs organized monthly groups centered on culturally informed themes or holidays for all transgender patients. Food was provided, and the first half hour was dedicated to socializing, followed by an icebreaker game, and a facilitated discussion about health and wellness.
**Community Events**	The PHEs organized larger culturally informed social events that focused on PrEP. These events included a PrEP Posada: a pageant show that advocated for PrEP within the transgender community, as well as pop-up photo booths and outreach events at Pride events. Additionally, larger outreach events were held to showcase the talents of participants at local Latinx nightclubs and bars.

### Selection and training of peer health educators (PHE)

The two PHEs were central to the Triunfo intervention. Both PHEs were trans Latinas, native Spanish speakers, and well respected and known within their communities. Both had expansive social networks and had been living in the Bay Area for decades. One of the PHEs had experience previously working with HIV programs at La Clinica. Both PHEs brought expertise in entertainment (i.e., shows, nightlife performances) and one had expertise in event production. The PHEs were selected because of their deep community connections, warm and outgoing personalities, commitment to wellbeing of their communities, and experience in HIV services.

Training for the PHEs occurred during an initial two-day training on integrated Next Step Counseling (iNSC) [32], a one-day training on the provision of trauma-informed services, and on the job training as needed. The two-day iNSC training included motivational interviewing, health education, intervention delivery, and the importance of boundaries. Examples of on-the-job training included ongoing education about relevant topics, such as new developments in PrEP provision and hormone therapy, and updated information about trans health issues.

### Sampling and recruitment

Study staff purposefully recruited interview participants (*n* = 13) from Triunfo with a range of engagement with PrEP and program services. Eligible participants were 18 years of age or older, HIV-negative at enrollment, and identified as a gender other than the sex they were assigned at birth. Bilingual co-author LV led recruitment for the qualitative interviews, approaching Triunfo participants via text or in the drop-in clinic to ask if they were interested in participating in an interview, and coordinated an interview appointment at a time and day of their convenience.

We also interviewed staff (*n* = 5) who were central to the Triunfo program experience. Since it was a relatively small project team, we began by interviewing all three project service providers: the clinician and both PHE. We added ancillary staff- a security guard and custodian- when it became apparent through interviews with Triunfo participants that the attitudes and behaviors of general clinic staff impacted participant experiences with the intervention. Additionally, the overall clinic environment is key to the patient experience, particularly when it comes to navigating the shared areas in the clinic, such as the entrance, bathrooms, pharmacy, and laboratory for blood draws. If a patient is misgendered upon entry or made to feel unwelcome, that patient could either not return or never get to their appointment with the clinician. Thus, speaking with staff in ancillary staff roles was an important part of understanding patient experience and the intervention.

### Data collection

Interviews were conducted in person at La Clínica, except for the PHE interviews, which took place at UCSF. Bilingual author SZH conducted interviews in either Spanish or English, depending on participant preference. Interviews with intervention participants lasted from 40 min to an hour, while interviews with staff lasted about 30 min. All interviews took place in a private office with respect for participant confidentiality. With participants’ permission, interviews were audio recorded. In two cases, we were not able to transcribe interviews (in one case due to the recorder not working and in the other the audio file was corrupted and could not be transcribed) and relied on detailed field notes that included an in-depth summary of the interview.

Semi-structured interview guides were tailored to either intervention participants or staff and further tailored for staff’s specific roles. The interview guides for intervention participants were initially developed in English and then translated to Spanish. Co-authors LGM, LV, CH, AS, and SZH (three native Spanish speakers, one bilingual person, and one who spoke moderate Spanish) reviewed the Spanish language guide as a group to edit for flow and resolve any issues with translation. Key domains of interest for intervention participants included awareness of PrEP (i.e. “tell me how you first heard about PrEP”), decision-making for PrEP initiation (“how did you decide PrEP was right for you?”), experiences taking PrEP (“tell me about your experiences on PrEP to this point, how’s it going?”) and general experiences receiving health care at the clinic (“What do you like best about receiving care at this clinic? What could be better?”).

The staff interview guide was developed in English and translated to Spanish, with two bilingual team members reviewing for accuracy. Four of the five staff interviews were conducted in Spanish. Domains of interest for key staff included descriptions of their role (“Describe your role on the Triunfo project?”), responsibilities (“Tell me about what that entails on a typical day?”), and general impressions on what was working well or could be improved with the project (“Now that the intervention is well underway, how is it going?”). For the PHE and clinician interviews, the interview guide was tailored to explore how and why the intervention was adapted to meet the needs of participants, such as inquiring into the development process for community events and expanding the navigation services to include legal appointments. The interview guide for ancillary staff had questions about how they were trained to work with trans populations, what their interactions had been like with program participants, and any other thoughts they wanted to share about Triunfo services.

Ethnographic observations were conducted over six months, during weekly clinics, monthly groups, and community engagement events. The observer was bilingual. Observations focused on staff interactions, dynamics between participants and intervention staff, topics of conversations during the drop-in clinic, monthly discussion sessions, and noting community event attendance and nature of participation.

### Data analysis

We conducted thematic analysis [33] of verbatim interview transcripts and used Dedoose to store and code data [34, 35]. Interviews were transcribed in their original language of data collection, using a professional transcription service. To stay as close to the participant’s words as possible, we leveraged our bilingual study team to code and conduct analysis in both English and Spanish. All quotes included in the findings section are in their original language of data collection, with English translation provided for quotes originally obtained in Spanish. Our team conducted translations of the Spanish quotes into English, with translations reviewed by three bilingual team members, including two native Spanish speakers and one native English speaker. Given that two members of the author team are English monolingual, one bilingual co-author (LV) who did not conduct the interviews engaged in a lengthy and intensive data familiarization process, translating all Spanish interviews into English. The translated transcripts were included in the data familiarization and immersion process when co-authors read and re-read transcripts.

We incorporated use of the field notes based on the unrecorded interviews as well as the observation field notes in the latter phase of data analysis. For the interviews, we compared the interview notes with the emergent themes to see if the information from these two participants aligned or contrasted with our findings from analysis of code reports. For the observation data, we used these data to elaborate on the overall clinic environment, both the environment inside the clinic and the surrounding areas. We relied on observational notes to enrich understanding of the group, drop-in clinic, and community engagement events, to add nuance to our descriptions interpersonal dynamics as well as culturally specific adaptations of the demonstration project.

We developed a codebook consisting of deductive codes (e.g., PrEP persistence, intervention experiences) that were of interest based on our aims and limited the number of codes and sub-codes to facilitate coding. To refine the codebook and ensure similar coding, two analysists - one who is a native Spanish speaker, one who is bilingual - separately coded the same transcript, and the met together to compare coded excerpts, clarify code definitions, and add or reduce codes as needed to ensure similar application and sufficient codes to capture the data. We then separately applied the revised codebook to a new transcript excerpt and compared to check code application and excerpt range concordance and were satisfied with the degree of shared application. We then coded all the transcripts with the revised codebook.

Once all interviews were coded, we entered a second phase of coding, where we read lengthy code reports such as “intervention experiences” aloud and summarized the data in brief notes. The goal of this phase was to connect descriptions of participants’ experiences and reflections back to the larger goal of understanding intervention implementation.

In our final phases of analysis, we engaged concept mapping to chart interrelationships between main themes and sub-themes and leveraged memos to develop analysis around the main themes. PHEs reviewed and discussed the charted themes and sub-themes with the larger analysis team as a form of member checking and to integrate their insights into the formal analysis [36]. These member checks were conducted over video chat, reviewing, and discussing the concept maps. Together with the PHEs, we prepared abstracts and presented preliminary analysis at conferences and scientific meetings, which served as the basis of this manuscript. Analysis took place over 18 months, through weekly group meetings and intensive presentation development meetings.

### Ethics

All study participants provided informed consent to participate in this study. The study protocol received ethics approval from the University of California, San Francisco Institutional Review Board (IRB). All methods were carried out in accordance with relevant guidelines and regulations.

## Results

### Clinic setting

The welcoming urban clinic is within walking distance of the Fruitvale Bay Area Rapid Transit District passenger station and multiple bus lines, and is located in a vibrant, predominantly Latinx immigrant community in Oakland, California. Fruit and flower stands are situated on sidewalks and along the street curbs outside of the main clinic. The surrounding neighborhood is densely packed with small businesses and shops catering to the needs of residents, from Mexican bakeries to shoe repair shops, primarily advertising in Spanish.

La Clínica has multiple physical locations in the neighborhood which specialize in different services, such as dental care and adolescent health. Triunfo was housed in the flagship location, a multi-storied building including emergency care, pharmacy, laboratory, and other specialty services. La Clínica staff are bilingual and reflect the patient population in terms of ethnic and racial background, culture, and immigration experiences.

### Description of participants

#### Intervention participants

We interviewed a total of 13 Triunfo intervention participants ranging in age from 18 to 44. Here we focus on interviews with trans Latina women (*n* = 11). While the overall set of interviews provided similar findings around program benefits, we chose to center the experiences of Spanish speaking trans Latinas, most of whom had immigrated to the US from Latin America, as the Triunfo intervention was tailored to this population. Intervention interview participants were mostly low income with limited access to education. Participants worked a range of jobs, such as hair styling, factory work, sex work, or driving for ride sharing companies like Uber. A few participants worked several part-time jobs, while struggling to earn enough to survive in the Bay Area. Employment, and thus income, was unstable for most. Many participants shared struggles with housing stability. Some were unhoused, staying with friends, or living in shelters. Those recently released from immigration detention (*n* = 3) lived with their sponsors. A few lived with romantic partners.

Many of the trans Latina intervention participants we interviewed shared intense experiences about past sexual abuse and rape, including childhood sexual abuse. A majority had survived intimate partner violence or transphobic attacks and harassment at school, from strangers, or at work. The recently arrived asylum seekers recounted their long cross-continental journeys by bus and by foot, filled with accounts of economic and sexual exploitation. Nonetheless, participants innovated and strategized to support themselves financially and sought out social support through community. Their stories attested to perseverance and a deep desire to live full, successful lives.

### Intervention staff

We interviewed five staff participants: the intervention clinician, the two peer health educators, one janitor, and one security guard. The intervention clinician was a bilingual cisgender woman, of white or Anglo-American ethnicity, and an experienced track record both providing gender affirming care and family medicine. The peer health educators were both transgender Latinas and native Spanish speakers. The custodian was a gay Latino, while the security guard was a straight Latino. Both were native Spanish speakers.

### Trusted space

Recruitment methods, clinic and study staff behaviors, and the overall clinic environment worked in tandem to create a welcoming, trusted space for study participants. PHEs, hired in part for their respected positions within Bay Area trans communities, led recruitment through their social circles and via community engagement events. PHEs approached friends in person, over text, and through social media posts to share information about the study and invite people for an initial eligibility visit.

Hearing about the study through friends (i.e., PHEs or other study participants) made participation more appealing and piqued curiosity about PrEP. Most study participants described initial caution or skepticism about PrEP and research studies. While a few had been on PrEP before Triunfo and at least one had participated in a previous PrEP study, most shared that hearing about the intervention through a friend – either a PHE or someone enrolled in the study - contributed to their decision to come into the clinic and anticipate trans competent services.

Mis amigas me decían que viniera, pero yo no quería venir…soy muy reservada…Tenía mucha depresión, no quería yo habe- venir. Y, cuando vine, me gustó cómo me trataron. Porque, antes uno compraba las hormonas y te las inyectara. No íbamos Bueno monitoreadas por una doctora… Vine [a Triunfo] y, wow, me gusto. Me encantó como me trataron las chicas en frente, la gente cuando entra uno aquí como es de amable con nosotros. Agarré mucha confianza. Porque, en toda mi vida, nunca he ido a un hospital ni a una clínica. Sí, hasta ahora, hasta este etapa de mi vida…Por falta de información, ignorancia, miedo. Todo. -Victoria, 40 años de edad

*My friends told me to come, but I didn’t want to… I’m very reserved… I had a lot of depression…when I came, I liked how they treated me. Because…before you bought hormones and injected them…we weren’t…monitored by a doctor… I came [to Triunfo] wow, I liked it. I loved how the girls in front treated me, the people when one enters here how friendly they are with us. I gained a lot of confidence. Because, in all my life, I have never been to a hospital or a clinic. Yes, until now, until this stage of my life…Due to lack of information, ignorance, fear. Everything. – Victoria, 40 years old (translated from original Spanish language quote)*

La mayoría todas las chicas nuevas…están llegando de otros países…ahorita tengo muchas que están llegando por asilo, todas esas chicas quizás vienen con otra mentalidad…veo como que no confían muy bien acerca de los servicios en las clínicas, porque quizás no han tenido buenas experiencias o no han tenido más bien, ninguna experiencia en la clínca, y se sienten…. He escuchado comentarios que dicen que nunca pensaron que las iban a tratar tan bien en una clínica…Desde el momento que entran a la clínica, desde la persona de seguridad que está en frente, las saluda a cualquier persona; las de limpieza, ahí todo mundo son muy amables con ellas. Ellas han tenido como una muy buena experiencia con toda la clínica.- Blanca, PHE

*Most all the new girls…are arriving from other countries…I have many who are arriving for asylum, all these girls perhaps come from their countries with a different mentality…I see how they don’t trust very well about the services in the clinics, because perhaps they have not had good experiences or they have not had any experience in the clinic, and they feel…. I have even heard comments that say they never thought they would be treated so well in a clinic…From the moment they enter the clinic, from the security person who is in front, they greet anyone; the cleaners, everyone there is very kind to them. They have had a very good experience with the whole clinic. -* Blanca, PHE *(translated from original Spanish language quote)*

As described in the quotes above, several participants noted that before enrolling in Triunfo they had never received care in a formal medical setting. Participants chose to stay out of care as a self-protective strategy to avoid anticipated discrimination due to transphobia, immigration status, and/or lack of health insurance. Others described previous negative experiences in medical settings, such as being misgendered or receiving poor quality medical services. Participants’ previous negative experiences and anticipated transphobia meant that many had taken hormones without medical supervision. Some participants noted that they were feeling better since they began receiving hormone therapy at the clinic with regular monitoring from the Triunfo clinician.

Las hormonas las he tomado hace muchos años…Ahora, estoy tomándolas más educadamente... Porque, antes tomaba hormonas que en veces te venden tus amigas, o las compras en la calle, o no sabes si te están haciendo un bien…Entonces, aquí en la clínica tienen que te llevan por un camino, que no te estén haciendo daño tus hormonas en tu riñón…porque las hormonas también causan daño. Entonces, aquí mantienen todo muy bien checada, y me tienen como que: ah, me están vigilando que no me vayan a hacer daño. Y, estoy más contenta aquí, porque me dan las hormonas que necesito, que son bien para mi cuerpo - Teresa, 29 años de edad

*I have taken hormones for many years… Now, I am taking them in a more informed way… Because, before I used to take hormones that sometimes your friends sell you, or you buy them on the street, or you don’t know, if they are doing you good ... So, here at the clinic they have to take you down a path, that your hormones are not hurting your kidneys…because hormones also cause damage. So, here they keep everything very well monitored, and they have me like: ah, they are watching me that [my hormones] are not going to hurt me. And, I am happier here, because they give me the hormones I need, which are good for my body – Teresa, 29 years old (translated from original Spanish language quote)*

Recruitment through social networks brought most participants in for their initial visit; however, the kind, respectful, and quality care they received at La Clínica kept them coming back. PHNs had recruited all but one of the interview participants, and she had learned about Triunfo from the program clinician with whom she had a history of receiving care. To ensure a welcoming and affirming experience, the intervention team trained La Clínica staff, from security guards to receptionists, on the fundamentals of creating a welcoming environment. Ground rules- such as using people’s preferred name rather than legal names if they differed and asking pronouns if unsure- helped participants feel welcomed from the moment they walked in the door, through registration, until making their way to the Triunfo intervention space.

The connection between…being in a clinic where the staff speaks Spanish…the overall institution is serving the Spanish speaking population…getting gender affirming care in that context, there’s added benefit. That helps shape people’s experience and people’s trust…Our frontline staff trainings have gone a long way…having our registration staff be familiar with the basics of name and pronoun and sort of legal changes to name and pronoun, and implications for medical documentation…all staff has some facility with navigating that and addressing patients appropriately. That cannot be overstated how critical it is. We’ve had registration staff, our front desk clerks, our pharmacy, our security guards, our lab, our medical assistants trained…by virtue of being here week after week, [trans patients] are constantly interacting with those departments, and so now there’s some practice under everyone’s belt – Aimee, clinican

Participants described feeling “en casa” and feeling warmly received.

Desde el primer día que llegas aquí el primer día que llegas aquí, te reciben como si estuvieras en casa. Te hacen sentir en confianza. Yo cuando vine, y fue en esta oficina, en esta misma oficina que me vine a escribir. Y, este, y yo- me daba pena por mi nombre que tenía antes, y me dijo- a mí me, me dijeron las chicas: “no, ya no te de pena, si yo me llamo así…. “que después te cambias el nombre y ya” - Victoria, 40 años de edad

*From the first day that you arrive here, the first day you arrive, they make you feel at home. They make you feel like you can trust them. When I arrived, I came to this office, this same office where I came to register, and I was uncomfortable about the name I had before, and they told me, the other girls told me, “Don’t worry, say I go by….and afterwards you can change your name and that’s it, done - Victoria, 40 years old (translated from original Spanish language quote)*

Interviews with clinic staff demonstrated an organizational commitment to offer welcoming services to trans people, whom they recognized as a vulnerable group. For lesbian, gay, or bisexual staff at La Clínica, the Triunfo program felt like a needed expansion of LGBT-specific services and a strong step forward to combat the homophobia and transphobia many described as entrenched in Latinx cultural norms. A janitor we interviewed, who shared that he had been rejected by his family for being gay, described the program as part of a step forward for equality, that in spite of discrimination people must “…salir adelante (*move forward/ progress*)” and that the program and peer educators provided “…un ejemplo importante de alguien que está viviendo tu vida actual, tu vida verdadero (*an example of someone living their real life, their authentic life*).

### Building psychosocial trusted space

The trusting relationships that Triunfo participants developed with intervention staff, including the study clinician and PHEs, proved the heart of the intervention’s success. Participants unanimously spoke highly of the study clinician, who has a long history of providing primary care to trans people and working in Spanish speaking immigrant communities. The clinician provided trauma-informed, patient-centered care that prioritized the participant’s sexual health needs and desired gender expression. She offered PrEP as one component of comprehensive sexual health care. Her strategies of asking questions, treating patients personably, and avoiding assumptions about patient preferences made space for patients to express their desires and share their experiences, without fear of judgment. In the quote below, a participant summarized appreciation of the clinician’s approach to collective decision making, a sentiment expressed by many.

La doctora me gusta, porque uhm, ella no toma las decisiones solamente, si no que me hace participar en las decisiones y me pregunta si está bien. Si no está bien, pues

ella respeta mi opinión. Y, este, y me dice lo que tenga que hacer y digamos, una comunicación muy bonita con la doctora, y me gusta. Igual que [mi terapista], porque a [mi terapista] le empecé a contar cosas que no, no le cuento a nadie, y es la única persona que le tengo confianza, a ella. Mm-hmm. Pero, sí. Me han ayudado bastante. Estoy bien agradecida, bien agradecida con la clínica – Victoria, age 40

*I like the doctor, because she doesn’t make the decisions on her own, she has me participate in decision making and asks me if things are ok. If its not ok, well, she respects my opinion. And she tells me what I have to do and we have beautiful communication and I like that. This is the same with [my therapist], who I have begun to tell things I have never told anyone else. She is the one person who I trust. So yes, they have helped me a lot. I am very grateful, very grateful for La Clínica.” - Victoria, age 40 (translated from original Spanish language quote)*

### Comprehensive patient navigation

#### Within clinic navigation

Led by PHEs, Triunfo provided comprehensive patient navigation for gender affirming care, ancillary health services within La Clínica, benefits and entitlements, and legal services. Initially, the PHEs focused on within-clinic navigation to ancillary services such as lab work, insurance support and registration, and pharmacy. They assisted participants to obtain benefits and entitlements, such as completing health insurance applications and unemployment insurance paperwork. These services were in different parts of the clinic than Triunfo and participants appreciated the companionship of the PHEs, who were bilingual, knew the other clinic staff, and were comfortable answering questions that the participants had. Particularly considering anticipated stigma, the presence of the PHE helped “las chicas” complete their initial blood draws and medicine pick-ups, which often required waiting in line with patients for urgent care or other health services. In the quote below, a participant compares her experience at Triunfo with a clinic elsewhere.

Acá es muy diferente. [Las PHE] me dicen un día exacto…ir y ya nos acompañan. E incluso hasta a la hora de ir al laboratorio nos acompañan. Nos llevan…abajo a sacar lo que son los análisis de sangre y están ahí junto con nosotras hasta que salimos. Esa es una experiencia muy bonita...Nos sentimos apoyadas –Christina, 35 de edad

*Here it is very different. [The PHE] tell me an exact day…and they already accompany us. And even when it comes to going to the laboratory they accompany us. They take us…downstairs to get blood tests and they are there with us until we get out. That is a very nice experience….We feel supported. -Christina, age 35 (translated from original Spanish language quote)*

#### Outside of clinic navigation

As intervention implementation progressed, Triunfo expanded navigation services in response to participant needs. PHEs assisted participants with transport to gender affirming care services not available at La Clínica, such as electrolysis or laser hair removal, an initial step for some gender affirming surgeries. PHEs additionally helped with transportation and paperwork completion for name changes and to obtain legal identification. For participants who had recently immigrated to the US, in many cases to seek asylum from transphobia and persecution in their countries of origin, PHEs drove participants to legal appointments and accompanied them for support as requested.

Las llevo a la corte a hacer el cambio de nombre, a nuestras chicas participantes, las que no tienen carro también antes de clínica paso a recogerlas a algunas que tienen dificultad para caminar o dificultad para llegar a la clínica, porque tenemos pacientes así…una de mis compañeras y yo nos turnamos para pasar por ellas. El trabajo de nosotros también es un poquito estresante, pero lo hacemos con mucha buena intención. Julia, PHE.

*I take our participating girls to the court to change their names, those who do not have a car, also before the clinic I go to pick them up some who have difficulty walking or difficulty getting to the clinic, because we have patients like that…one of my co-workers and I take turns picking them up. Our work is also a little stressful, but we do it with a lot of good intentions. - Julia, PHE (translated from original Spanish language quote)*

PHEs responded to patients’ most pressing needs, building a sense of trust with participants, who knew they could rely on staff for support beyond issues specific to PrEP. PHEs received phone calls after hours and on weekends about a range of acute daily life stressors, such as participants who were worried about being evicted, unexpectedly had nowhere to sleep that night, or had gotten into a bad fight with their boyfriend. Participants called in crisis, for example reaching out after having been assaulted by a sex work client, asking for advice and assistance on navigating post-rape care. Providing broad and consistent support, rather than focusing narrowly on HIV prevention, contributed to the success of Triunfo in initiating and retaining participants in care.

#### Navigating daily life: cross cultural needs and general advice

PHEs not only helped participants navigate care systems and personal crisis, but they also offered cultural navigation, as Latina immigrants themselves who had lived long term in the US. They shared lessons learned on how to get things done in this country. PHEs provided translation, explanation, and guidance.

Fuera de la clínica, es más como también llevarlas a hacer cambio de nombre, gender changes, papeles. Algunas que tienen problema con el idioma trato de ayudarles a traducer…o llevarlas a lugares donde ellas no sepan cómo llegar, cómo agarrar el seguro social o recomendarlas con algún lugar de migración y generalmente a veces yo las llevo con los abogados. Las chicas muchas están recién llegadas de sus países y no tienen o no saben cómo movilizarse aquí en este país. Ese sería más como mi rol, estar más tiempo con ellas, ayudarlas más – Blanca, PHE

*Outside the clinic, it’s more like taking them…to do name changes, gender changes, paperwork. Some who have a problem with the language I try to help them translate…or take them to places where they don’t know how to get there, how to get social security or refer them to a migration place and generally sometimes I take them to see lawyers. Many of the girls have just arrived from their countries and don’t have or don’t know how to get around here in this country*. -Blanca, PHE *(translated from original Spanish language quote)*

PHEs also supported participants’ holistic needs and offered social support that went beyond PrEP uptake and adherence, which benefitted participants regardless of immigrant background or language abilities. PHEs provided informal one-on-one peer counseling to participants on request during clinic hours. During these sessions, participants would express concerns around employment or workplace transphobia, share painful exchanges from their childhood or current interactions with family, and ask overarching questions such as how to talk with people about hormone therapy and resulting physical changes.

Para mí es muy interesante las necesidades de nosotros, especialmente para las latinas, cómo podemos lidiar con nuestras familias. Vienen y quieren empezar las hormonas, quieren ser ellas, quieren ser las mujeres que nosotros somos, queremos ser libres, entonces es muy difícil empezar-“¿Y cómo le voy a decir a mi familia que estoy tomando hormonas? Yo no les puedo decir que estoy tomando PrEP ni puedo decirles que el PrEP es un medicamento para prevenir VIH porque no quiero que vayan a pensar mal de mí”…Entonces, es como muy importante hacerles saber que no están haciendo nada malo, solamente estamos protegiendo y estamos protegiendo su salud y que ellas deben de ser quienes son. – Julia, PHE

*For me, our needs are very interesting, especially for Latinas, how do we deal with our families? They come and they want to begin hormones, they want to be themselves, they want to be the women that we are, we want to be free, and so it’s difficult to begin, “ and how will I tell my family that I’m taking hormones? I can’t tell them that I’m taking PrEP nor can I tell them that PrEP is a medication that prevents HIV because I don’t want them to think bad of me…So it’s very important to let them know that they aren’t doing anything wrong, we are protecting ourselves and we are protecting our health and they should be who they are. -Julia, PHE (translated from original Spanish language quote)*

In this way, PHEs responded to the most immediate needs of clients, helping clients feel valued and building trust that they mattered beyond the proximal concerns of the research study. By responding to what the participant determined as most critical, the intervention staff helped the participant make room for PrEP in their lives, as more pressing needs were also addressed.

### Social support “Entre Nosotras”

Social support “entre nosotras” (between us girls/women), laid the foundation for the success of Triunfo. Social support came from a variety of sources: participant interactions in the waiting room, group monthly meetings, community engagement events, and interactions with PHEs. While some of the participants already knew each other, the program provided a unique and distinct type of social support that centered on self-care, empowerment, and well-being. Several participants commented that outside of Triunfo, interactions between *las chicas* can be defensive or competitive. This differed from the way that participants supported and built each other up as part of Triunfo.

Aquí venimos para apoyarnos entre nosotras, le digo…antes no era así. Nos criticábamos, nos lastimábamos y ahorita ya todo cambió. Le digo, ahorita nos tenemos que apoyar entre nosotras….Yo era muy , este, estaba muy a la defensiva. Si me miraban así, yo: qué me miras. Y, luego echando así. Y, ya como cambié con las, uhm, con esto [programa]… cuando uno viene aquí, se siente que quieres venir - Victoria, 40 años de edad

*We come here to help each other…and I’m telling you, before it wasn’t like this. [Trans women] criticized each other, we hurt each other. I was always on the defensive. If they looked at me like this, I would say: what are you looking at? And it would go on like that. With this program I have already changed to much…when [you] come here, [you] can tell that they want to be here. – Victoria, 40 years old (translated from original Spanish language quote)*

#### Social support space: drop-in clinic

Over the course of the intervention, the waiting room, the dedicated Triunfo space at La Clínica, became a drop-in center. Located near the urgent care waiting room, the Triunfo room was bare, with no windows, bright white walls and florescent lights, a sink, an exam table, a table, and some folding chairs. Each clinic day, study staff laid out water bottles, a large bowl of condoms and set up chairs around the table. Participants and staff would bring food to share- fresh fruit with chile from the street vendors outside, pan dulce and coffee, sandwiches, or a home cooked delicacy like tamales. While participants would wait to see the clinician or to meet with staff to enroll in the intervention or complete another study activity, others would gather on days when they had no appointment, dropping in for “el chisme,” to gossip or share stories, to chat, to laugh and socialize.

Ya pues, salgo yo, hago lo que tengo que hacer, voy a mis compras. Y, a veces, cuando me siento enojada, yo de malhumorada, para no de contagiarlo del coraje de uno, de rutina, pues salgo a dar una vuelta a ver a mis amigas. Me vengo a la clínica aquí, y así me la paso, tranquila. Ya llego relajada y contenta- hoy, fíjate, me pasó esto, conocí una amiga nueva y todo. Y, platico con [ella] y estamos riéndonos, y [ella] como si nada….- Clarisa, 44 años de edad

*Well then, I go out, I do what I have to do, I go shopping. And sometimes, when I’m feeling angry or I am in a bad mood, but I don’t want to take it out on anyone….well I stop by to see my friends. I come to the clinic here and it passes, I feel calm. I already feel relaxed and happy. Today, for example, this happened to me and I met a new friend and everything. I spoke with her, we were laughing, and now it’s like nothing [was wrong] – Clarisa, 44 years old (translated from original Spanish language quote)*

The energy of the participants, the warmth of the PHEs, and the sounds of talking and laughter transformed the space into a different environment, a shared, trans space centered on immigrant experience, where people let down their guard, discussed whatever was on their minds, and returned to their individual lives feeling bolstered. Conversation flowed easily, generally keeping a light and playful tone, although topics ranged from beauty regimes to tips on seeking asylum. Clients talked to each other in affectionate terms (“hola princesa” “que hermosa” “te ves bonita”). While PrEP and hormone therapy were provided, the sense of group cohesion was a draw that brought participants in even on days they had no scheduled appointments.

The PHEs orchestrated an environment and culture of comfort, kindness, and enjoyment. They set the tone for the caring interactions and were always ready to listen to whatever participants wanted to share. The PHEs often went out to coffee or dinner with participants at the end of clinic days, which one PHE described as “creando un vinculo mas fuerte entre nosotras/ *creating a stronger bond between us*” making the group feel “mas unidas/ *more united*.” Below, the same PHE elaborates, sharing that the most important part of her job is to make the clients feel heard, even if she’s not “totalmente capatizada/ totally qualified,” like a therapist or psychologist, she does her best to listen and support.

Al llegar a la clínica queremos ofrecerles como un buen servicio y siempre estar amables con ellas, tratar de hablarles de cosas que tenemos en común o tratar de ayudarlas y entenderlas en lo que más podamos. Generalmente, algunas vienen con…problemas con su pareja o en su trabajo o que no tienen trabajo… mi deber más que nada escucharlas, quizás a lo mejor no estoy como totalmente capacitada para darles un consejo… pero creo que con escucharlas a veces ellas se sienten un poquito más reconfortables [sic]- Blanca, PHE

*When they arrive at the clinic, we want to offer them a good service and always be friendly, try to talk to them about things we have in common or try to help and understand them as best we can. Generally, some come with…problems with their partner or at work or they don't have a job…my duty more than anything is to listen to them… I am not totally qualified to give them advice …but I think that by listening to them, sometimes they feel a little more comfortable- Blanca, PHE (translated from original Spanish language quote)*

#### Individual, group, and community support

In addition to the clinic days, with their one-on-one sessions and group conversations, monthly groups and community engagement events provided additional opportunities to offer social support to participants. Monthly groups varied in size and were drop-in, one Friday evening per month. Generally held in the cozy front room of Trucha, La Clínica’s HIV prevention center, the groups began with a shared meal sometimes informed by a culturally relevant holiday and then proceeded to a rotating topic that reflected a key issue for participants. Groups were semi structured in that PHEs dedicated at least 20 min of conversation to PrEP and safe sex practices in the form of discussions, games, and anecdotal story sharing. Often a guest speaker would join, such as a medical expert on gender affirming surgeries or a lawyer who specialized in asylum cases. The groups would sometimes be outdoors; for example, PHEs might host a *carne asada* (BBQ) in the park or organize a soccer game.

As part of efforts to build social support and promote PrEP awareness, Triunfo facilitated several larger community engagement events. Around the winter holidays Triunfo hosted a PrEP Posada, a take on a traditional Latin American holiday celebration, with performances and dancing. Triunfo hosted an annual “Miss Triunfo” pageant, with program participants competing like a typical beauty contest, but with questions centered around PrEP and safe sex practices. The winner, Miss Triunfo, was selected based on her ability to serve as a community PrEP educator and represent the program in other Latinx and LGBTQ events, such as pride events. The groups and events helped to encourage a sense of community and reinforced the empowering, wellness centered messages that encouraged participants to live their best lives.

## Discussion

Beneficial elements of the Triunfo intervention at La Clínica centered around three intertwined themes: creating trusted space, providing comprehensive patient navigation, and offering social support “entre nosotras” (“between us women/girls”). The combination of these three factors contributed to the intervention’s success supporting participants to initiate and persist on PrEP. The PHEs directed implementation of the intervention components, trained clinic staff on providing trans competent services, and set the tone for a friendly and welcoming dynamic for study participants. Together, with the gender affirming intervention clinician, the PHEs fostered a healing space, where participants could share challenging experiences and seek solace or share laughs and enjoy a mid-day snack. Our findings demonstrate that to successfully co-locate gender affirming care with PrEP services, PrEP provision was but one component of a broader, more holistic program in the context of a community-based clinic with extensive culturally specific expertise and responsive services.

Findings from our qualitative evaluation of Triunfo, a demonstration project that co-located gender affirming care and PrEP services at La Clínica de Raza in Oakland, show the power of a Latinx centered, holistic, welcoming clinic environment, combined with compassionate and trans competent clinical and ancillary staff, to overcome barriers to care and successfully meet the sexual health needs of trans Latinas. PHEs leveraged their social capital, empathy, and personal experience to recruit participants for the program and support their retention in care. While an ample body of evidence shows the value of peer navigation in HIV services [37–40], our research adds nuance to the existing literature by profiling the multifaced roles that PHEs served for primarily Latina, transgender, immigrant participants; addressing their needs for gender affirming care, information around immigration, and bilingual services from primarily Latinx staff. Treatment of participants by the PHEs as well as the Triunfo staff and the program clinician modeled a way of caring that set the tone for the program, creating a climate that provided a gender affirming experience from the moment patients entered the clinic, and carried through their clinical consults. The PHEs, the Triunfo staff, and the Triunfo clinician played crucial roles in introducing refinements to the program and content that ensured ongoing responsiveness to participant priorities.

Our research contributes to the call for services that address the intersectional experiences of Latina trans women in HIV programming [41–43]. Participants’ identities as heterosexual women (with one exception, who identified as pansexual), transgender Latinas, in many cases immigrants and Spanish speaking, were all integral to their lived experiences. Triunfo respected and affirmed these identities during interactions with staff and in the range of services provided. Participants formed close bonds with each other, with the PHEs, and the clinician that contributed to a sense of wellbeing and trust “entre nosotras,” echoing the social support networks documented in earlier research with trans Latinas [44]. Triunfo’s full range of programming connected participants to gender affirming care and immigration services available in the Bay Area. Findings demonstrated that HIV prevention services with a client-centered approach that responded to other competing needs successfully engaged Latina trans women, many of whom had never received medical care in a clinical environment before Triunfo.

While the Triunfo clinician (also the site PI on TRIUMPH) brought extensive expertise in providing HIV and gender-affirming care to trans Latinas and was the project champion to establish a new line of gender affirming services at La Clinica, educating the rest of the staff in creating a welcoming environment proved another lesson from Triunfo. Multiple participants cited the importance of being treated with respect from the moment they walked into the clinic; for example, registration and security staff used correct pronouns and made participants feel welcome. These findings reinforce evidence from other health services research with trans populations, that using correct pronouns and preferred names is fundamental to communicating respect for trans people in clinical settings [45–47]. Our findings highlight the need to build trust with trans participants; as many participants shared, they had avoided care in the past due to anticipated stigma.

Given higher rates of HIV among trans women as compared to trans men and nonbinary individuals [4], TRIUMPH focused on recruiting and enrolling trans women. Trans Latino men and other gender expansive people may have different needs from a comprehensive sexual health program, including PrEP, depending on their sexual behaviors and desires [13, 48]. A PrEP or HIV services program geared towards trans men, nonbinary, and/or gender expansive people would likely need to be structured differently, such as including peer navigators who are trans men and nonbinary people and creating a waiting room and social support network centered on trans men’s, nonbinary, and/or gender expansive people’s experiences.

While the comprehensive patient navigation proved an important part of the program, integrating additional mental health services and case management are crucial areas for additional implementation research. Participants shared past experiences with trauma and acute ongoing needs due to experiences with transphobia, violence, and discrimination, like findings from research in other contexts [40, 49, 50]. While the PHEs provided peer support and the clinic offered some case management and mental health care, this patient population shoulders high level of need and could benefit from additional services. La Clínica has one full time therapist for all patients, and anecdotal experiences from the program point to a lack of Spanish speaking mental health care providers in the Bay Area. Future programmatic efforts should integrate additional training for PHEs in the provision of mental health support and case management for trans women and other populations experiencing marginalization [51]. Another key area for implementation research is how to integrate additional support for PHEs, including clinical supervision, into programs that rely on PHEs to address vicarious trauma and secondary traumatic stress experienced by the PHEs themselves [52].

## Conclusions

Our qualitative evaluation of a PrEP demonstration project tailored for trans Latinas living in the San Francisco Bay Area showed that addressing their multifaced needs was pivotal to the success of the program. The program layered a variety of social support and community engagement mechanisms with patient navigation to PrEP and gender affirming medical care. The clinician and PHEs invited open, honest conversations about sex, intimacy, and priority concerns from all areas of participants’ lives. This holistic approach, combined with the welcoming and trans competent clinic environment with extensive cultural expertise, engaged a population with little to no experiences receiving care in a medical setting, yet had high unmet needs.

## Data Availability

The datasets generated and analyzed during the current study are not publicly available, as we did not receive participants consent to share their transcripts outside of the study team. Further, while de-identified, the transcripts contain personal and sensitive narratives that touch on sexual and physical violence, undocumented immigration, and drug use, that could be identifiable to people who know them well (i.e., abusive partner). Data are available from the corresponding author on reasonable request.

## Footnotes

1 Of note, we use the term “Latinx” as a gender inclusive term to refer to people from Latin American or Caribbean countries of origin- including immigrants and second or third generation immigrants- living in the U.S., to challenge notions of gender binary language and recognize the intersectionality of gender, language, and culture14.Salinas C, Lozano A. Mapping and recontextualizing the evolution of the term Latinx: An environmental scanning in higher education. Critical Readings on Latinos and Education: Routledge; 2019. p. 216 − 35.
